# Distribution of dental practices in Jazan of Saudi Arabia: a GIS-based approach

**DOI:** 10.1186/s12913-023-09337-3

**Published:** 2023-04-11

**Authors:** Mosa Ali Shubayr, Estie Kruger, Muhammad Mansoor Majeed, Afrah H Hattan, Shoaa Ahmed Jearan, Marc Tennant

**Affiliations:** 1 International Research Collaborative, Oral Health and Equity, School of Human Sciences, The University of Western Australia, Nedlands, Australia; 2 Department of Preventive Dental Sciences, College of Dentistry, Jazan University, Jazan, Kingdom of Saudi Arabia; 3 Department of Oral Biology, Altamash Institute of Dental Medicine, Karachi, Pakistan; 4 International Private Clinic, Abha, Aseer, Kingdom of Saudi Arabia

**Keywords:** Accessibility, Dental healthcare services, Health facilities, Geographic information systems, Jazan, Kingdom of Saudi Arabia

## Abstract

**Background:**

Jazan region in the Kingdom of Saudi Arabia (KSA) has been extensively studied regarding access to dental care services, but there is currently no specific study on the distribution of public (primary healthcare centres (PHCs) and hospitals) and private dental healthcare facilities in the area. This study aimed to evaluate the spatial distribution of public and private dental care facilities in the Jazan region in relation to the population distribution in each governorate of the region.

**Methods:**

The most up-to-date, easily accessible, and anonymous data and information were used for this investigation. The Ministry of Health’s (MOH) Statistical Yearbook 2020 and interactive map were used to identify the locations of healthcare facilities. These locations were plotted on a map using Google Maps, and the data was converted to longitude and latitude with 90% level building accuracy. QGIS’s integrated database was used to develop buffer zones and perform attribute analysis. The data was then exported for analysis in Microsoft Excel, where healthcare facility-to-population ratios were determined.

**Results:**

In Jazan region, consisting of 17 governorates and a population of 1,726,739, there were 275 public and private dental clinics, with a ratio of one dental clinic per 6,279 people in terms of general health services. Only 12.4% of these clinics were located beyond 20 km of the city centre, serving approximately 70% of the region’s population.

**Conclusion:**

The uneven distribution of dental clinics in the Jazan region has hampered access to dental treatments and has led to a significant burden on dental health facilities, reducing the quality of care available in the region. Mapping the distribution of MOH, private, and other health facilities, as well as the burden of oral disease in the Jazan region, is necessary for further research.

## Introduction

The availability of healthcare services is an essential component of a good quality of life [1]. However, ensuring equal access to healthcare services can be challenging due to various constraints such as economic, political, and geographical issues [1]. Access to healthcare is defined as the ability to use personal health services in a timely manner to achieve optimal health outcomes [2]. In many cases, the terms access and utilization are used interchangeably [3]. Dania and Frasi define access to dental healthcare services as the availability and use of care [4]. According to Penchansky and Thomas, access to healthcare refers to the degree to which the healthcare system aligns with the needs of the patient [5].

In KSA, dental caries and periodontal diseases are the most common oral diseases [6]. The prevalence of caries in the country is high, with estimates ranging from 59 to 80% in children. A study conducted in 2014 found that every participant in a population aged 18–40 years old had gingivitis, indicating a prevalence of 100% for this age group [7].

Various methodologies have been used in studies on access to healthcare services, including Geographic Information Systems (GIS), systematic reviews, cross-sectional studies, and questionnaires [8]. Alsharif states that GIS utilizes various scales to assess and measure various correlations between spatial and temporal trends of healthcare-related variables, population, and environmental risk factors [9]. Understanding the distribution of healthcare services is important for addressing healthcare disparities and improving access to healthcare services, according to Alonge and Peters [10]. In Jazan region, several factors limit the expansion and improvement of oral healthcare services, with the most significant being the distribution of dental service providers and practitioners. This helps explain why dental care in Jazan region is less accessible and of lower quality than in other parts of the country [11].

Studies on the geographic accessibility or factors affecting the distribution of dental healthcare services among the regions of the KSA are scarce. Studies using GIS to quantify accessibility to primary and private healthcare services in the Jeddah region have found that some areas, such as the northern and central districts, have limited or inadequate access to available healthcare facilities [12–14]. Alsharif’s evaluation of primary and private dental practices in Al Madina, KSA, also found that there were more dental facilities in the city centre but relatively fewer in outlying areas [9]. According to a study, there is a shortage of dental healthcare services in Jazan. The uneven distribution of health facilities reduces the region’s service quality [15]. Shubayr et al., has provided a description of the public dental facilities in the Jazan region, KSA and concluded that accessibility issues restrict people from receiving necessary healthcare [15]. The study also highlighted the importance of additional research to identify public and private dental facilities in the Jazan region.

To the best of the researchers’ knowledge, no prior studies have examined the geographic distribution of public and private healthcare services in Jazan region. This study aims to fill this gap in the literature by analysing the spatial distribution of dental clinics and healthcare facility-to-population ratios in the region. The results of this study will provide valuable insights and recommendations for improving oral healthcare services in the Jazan region in the future.

## Materials and methods

Data for this study were collected between May 1st and August 30th, 2022. Ethical approval was not required since the data used in this study was publicly accessible and anonymous. Jazan region, which has the highest population density in the KSA, covers an area of 11,671 km2 [16] and is divided into 17 governorates. This study analysed the distribution of dental clinics in each of these governorates.

### Locations of dental clinics

The locations of dental clinics were sourced from the MOH Statistical Yearbook 2020 [17] and interactive map [18]. University dental schools, mobile dental clinics, and duplicate addresses were not included in the dataset. The focus of this study was on public (PHCs and Hospitals) and private dental clinics, and a total of 275 dental clinics were included in the analysis. The locations of 90% of these clinics were converted to longitude and latitude using Google Maps.

### Population statistics

Population statistics were obtained from the General Authority website [19] and the World Population website [20]. Administrative governorates were used as the geographical region for analysis, as this was the only data source available in a file format that was compatible with the population data files.

### Geographic integration

Quantum Geographic Information Systems (QGIS) was used for geographic mapping in this study (version 3.20, Essen, Germany). The World Geodetic System 1984 (WGS 84) was used for coordinate referencing, and all geographical and related population data were imported and analysed in Microsoft Excel (version 14.0; Microsoft, Redmond, WA, USA).

### Mapping

Random points in QGIS were used to display the coverage area for the identified dental clinics in relation to the target Jazan population. The study examined the accessibility of 275 dental clinics at four different levels (radius) using Quantum GIS to plot dots for the population on the study map. The main difference between these levels of accessibility (radius) was the distance from the centre of the governorate. Four buffer zones (5, 10, 15, and 20 km) were identified and analysed around the centre of each governorate. The number of healthcare facilities per person was calculated using census data.

The authors considered multiple approaches to determine the radii of the buffer zone from the centroid. For example, Mazen et al. used buffer zone radii of 2.5 km, 5 km, and 7 km to assess spatial accessibility to hospitals in Makkah, KSA [21]. Alsharif in 2020 used buffers with radii of 3 to 5 km as proxies for driving distances [22] to understand the geographic distribution of dental healthcare facilities in Al Madina, KSA [9]. In this study, buffer zones with incremental radii from 5 to 20 km were used.

### Statistical analyses

Data from this study were analysed both qualitatively and quantitatively using Microsoft Excel (2016 version 14.0). The ratios of dental healthcare facilities to the study population were calculated by transferring the data from the integrated database in QGIS to Microsoft Excel software. The distribution of the population was determined based on the population count database in the Worldpop.org database [20], which provides the population count for each grid cell in the database. The buffer zones were used to assess the variation in the distribution of dental clinics and population.

## Results

**Fig. 1:**
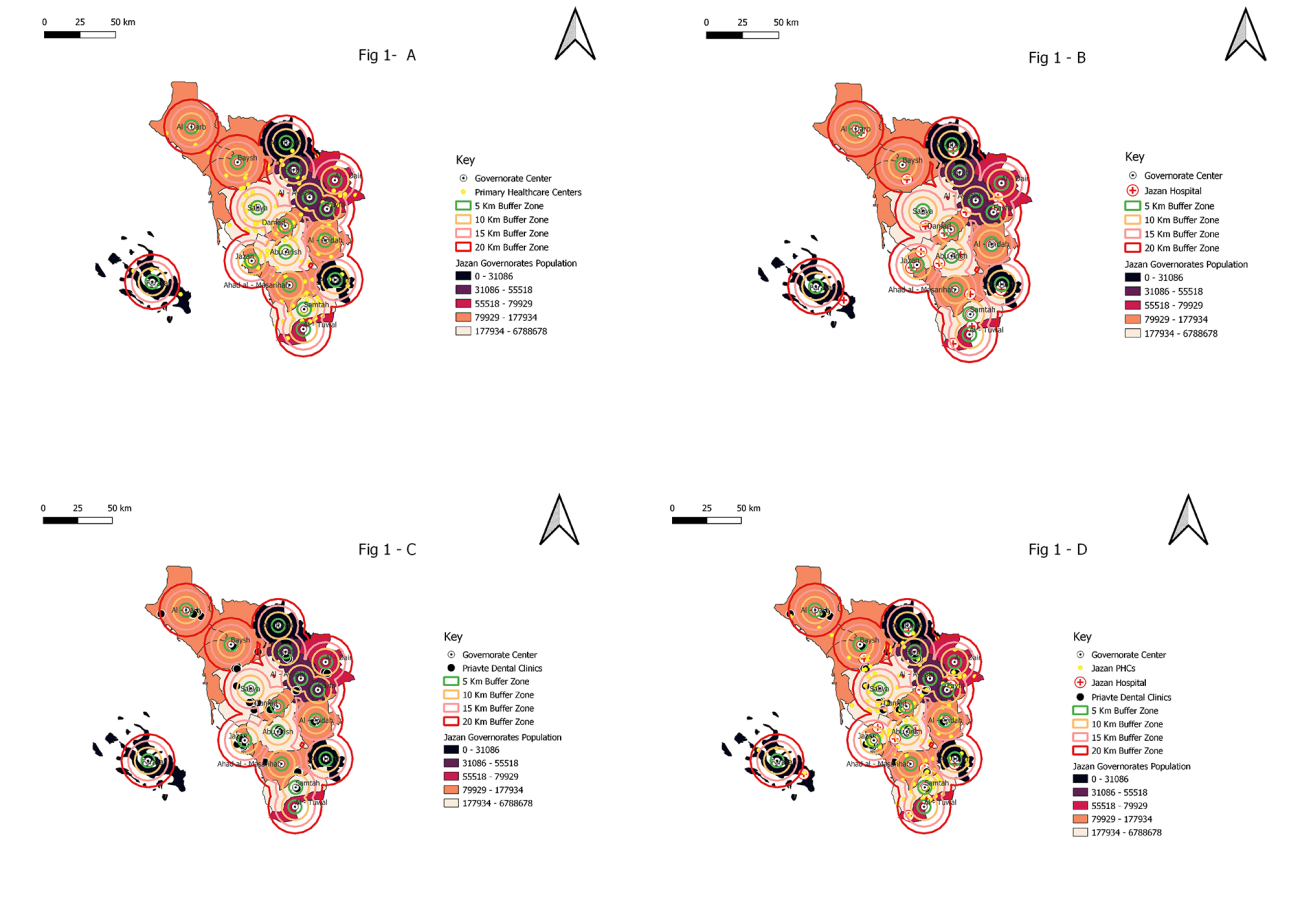
A high-resolution image showing the distribution of population per governorate and dental practice based on accessibility areas in the Jazan region, KSA. (a) buffer zone indicating primary healthcare centres (PHCs) distribution from governorate centroid (b) buffer zone indicating hospitals distribution from governorate centroid count (c) buffer zone indicating private dental clinics distribution from governorate centroid (d) buffer zone indicating all dental clinics distribution from governorate centroid

Jazan region, also known as Jizan, Gizan, or Gazan, consists of 17 governorates (mohafadat in Arabic) with a total population of 1,726,739 as of 2020. The majority of the region’s residents live in the major cities of Sabya, Abu Arish, and Jazan, with 290,244, 248,654, and 172,269 residents, respectively. A total of 275 dental clinics were geocoded by QGIS (Fig. 1).

Jazan, Sabya, and Abu Arish had the highest number of dental clinics, representing 18.2%, 14.9%, and 12.0% of the total, respectively (Table 1). However, many of the mountain and peripheral areas, such as Al Harth, AlRayth, Farasan, Al Aydabi, Altuwal, Harub and Fayfa, have limited dental clinics serving their populations range from 1.1 to 2.9%, and some of them, such as Al Harth, Fayfa, Altuwal, and Farasan, did not have any private dental clinics. Hospitals with dental clinics were more evenly distributed among the governorates throughout the region, with the percentage ranging from 5.6 to 11.1% (Table 1).

**Table 1:** Dental Practice -to-Population in Jazan Region, KSA

Items	Governorate	Population (%)	PHCS n (%)	Hospitals (%)	Private Clinics n (%)	Dental Clinics n (%)	Dental Clinics to Population Ratio
1	Jazan	172,269 (10.0)	13 (9.0)	2 (11.1)	35 (31.3)	50 (18.2)	1: 3,445
2	Sabya	290,244 (16.8)	21 (14.5)	1 (5.6)	19 (17.0)	41 (14.9)	1: 7,079
3	Abu Arish	248,654 (14.4)	18 (12.5)	2 (11.1)	13 (11.6)	33 (12.0)	1:7,535
4	Samtah	186,476(10.8)	18 (12.5)	1 (5.6)	10 (8.9)	29 (10.5)	1: 6,430
5	Al Harth	23,075 (1.3)	4 (2.8)	1 (5.6)	0 (0.0)	5 (1.8)	1: 4,615
6	Damad	90,827 (5.3)	7 (4.8)	1 (5.6)	3 (2.7)	11 (4.0)	1: 8,257
7	Ar Rayth	23,543 (1.4)	2 (1.4)	1 (5.6)	0 (0.0)	3 (1.1)	1: 7,848
8	Baysh	89,219 (5.2)	9 (6.2)	1 (5.6)	9 (8.0)	19 (6.9)	1: 4,696
9	Farasan	22,556 (1.3)	3 (2.1)	1 (5.6)	0 (0.0)	4 (1.5)	1: 5,639
10	Ad Dair	73,087 (4.2)	9 (6.2)	1 (5.6)	3 (2.7)	13 (4.7)	1: 5,622
11	Ahad Al Masarihah	139,498 (8.1)	12(8.3)	1 (5.6)	6 (5.4)	19 (6.9)	1: 7,342
12	Al Aydabi	40,106 (2.3)	4 (2.8)	1 (5.6)	3 (2.7)	8 (2.9)	1: 5,013
13	Al Aridah	96,420 (5.6)	8(5.5)	1 (5.6)	3 (2.7)	12 (4.4)	1: 8,035
14	Ad Darb	87,932 (5.1)	3(2.1)	1 (5.6)	6 (5.4)	10 (3.6)	1: 8,793
15	At Tuwal	68,760 (4.0)	5(3.4)	1 (5.6)	0 (0.0)	6 (2.2)	1 : 11,460
16	Harub	36,068 (2.1)	6 (4.1)	0 (0.0)	2 (1.8)	8 (2.9)	1: 4508
17	Fayfa	38,005 (2.2)	3 (2.1)	1 (5.6)	0 (0.0)	4 (1.5)	1 : 9,501
Total	1,726,739 (100)		145 (100)	18 (100)	112 (100)	275 (100)	1: 6,279

Abbreviation: PHCs: Primary Healthcare Centres, n: number

The region shows an overall practice -population ratio of 1 dental clinic to 6,279 residents. Jazan governorates had the lowest number of PHCs, hospitals and private dental clinics to population ratio (1: 3,445) and were one of the most populated governorates. On the other hand, mountain areas such as At Tuwal and Fayfa had the highest dental clinic to population ratios with 1: 11,460 and 1: 9,501, respectively (Table 1).

The majority of dental clinics (33.5% and 32.0%) in the Jazan region are located within 5 km and 15 Km of the centre of each governorate. However, only 3.3% and 17.7% of the population have access to healthcare services within 5 and 15 km, respectively. Only 12.4% of the dental practices, which served approximately 70% of the population, were located more than 20 km away (Table 2). The study shows that majority (39.3% and 38.9%) of PHCs and hospitals located with 15 Km, but more than half (51.8%) of private clinics located with 5 km from the governorates’ centres.

**Table 2:** Population Distribution Based on Buffer Zone in Jazan Region

Items	Governorate	Total Pop	Distance from Dental Centre (KM)	Population (%)	PHCs n (%)	PHC to Population Ratio	Hospital n(%)	Hospital to Population Ratio	Private clinics no (%)	Total no (%)	Total to Population
1	Jazan	172,269	0–5	11,422 (6.6)	7 (53.8)	1: 1631	1(50.0)	1:11,422	28(80.0)	36 (72.0)	1 :317
			6–10	32,175 (18.7)	4(30.8)	1:8,043	0(0.0)	0: 32,175	7(20.0)	11 (22.0)	1:2,925
			10–15	42,124 (24.5)	2(15.4)	1:21,062	1(50.0)	1: 42,124	0(0.0)	3 (6.0)	1:14,041
			> 20	86,548 (50.2)	0(0.0)	0 : 86,548	0(0.0)	0: 86,548	0(0.0)	0 (0.0)	0: 86,548
2	Sabya	290,244	0–5	1,705 (0.6)	2(9.5)	1:852	0(0.0)	0 : 1,705	0(0.0)	2 (4.9)	1:853
			6–10	5,083 (1.8)	2(9.5)	1: 2,541	1 (100.0)	1:5,083	0(0.0)	3 (7.3)	1:1,694
			10–15	14,213 (4.9)	11(52.4)	1: 1,292	0(0.0)	0 : 14,213	16(84.2)	27 (65.9)	1:526
			> 20	269,243 (92.8)	6(28.6)	1:44,873	0(0.0)	0 : 269,243	3(15.8)	9 (21.9)	1:29,916
3	Abu Arish	248,654	0–5	8,952 (3.6)	3(16.7)	1:2,984	0(0.0)	0 : 8,952	13(100)	16 (48.5)	1 :560
			6–10	16,973 (6.8)	4(22.2)	1: 4,243	1(50.0)	1:16,973	0(0.0)	5 (15.2)	1: 3,395
			10–15	30,692 (12.3)	9(50.0)	1:3,410	1(50.0)	1:30,692	0(0.0)	10 (30.3)	1 : 3,069
			> 20	192,037 (77.2)	2(11.1)	1:96,018	0(0.0)	0 : 192,037	0(0.0)	2 (6.1)	1 : 96,019
4	Samtah	186,476	0–5	13,134 (7.0)	4(22.2)	1:3,283	1(100.0)	1:13,134	10(100)	15 (51.7)	1:876
			6–10	38,066 (20.4)	6(33.3)	1:6,344	0(0.0)	0 : 38,066	0(0.0)	6 (20.7)	1 : 6,344
			10–15	64,756 (34.7)	5(27.8)	1:12,951	0(0.0)	0 : 64,756	0(0.0)	5 (17.2)	1 : 12,951
			> 20	70,520 (37.8)	3(16.7)	1:23,506	0(0.0)	0 : 70,520	0(0.0)	3 (10.3)	1 : 23,507
5	Al Harth	23,075	0–5	1,383 (6.0)	1(25.0)	1:1,383	1(100.0)	1:1,383	0(0.0)	2 (40.0)	1: 692
			6–10	4,809 (20.8)	2(50.0)	1:2,404	0(0.0)	0 : 4,809	0(0.0)	2 (40.0)	1 : 2,405
			10–15	16,698 (72.4)	1(25.0)	1:16,698	0(0.0)	0 : 16,698	0(0.0)	1 (20.0)	1 :16,698
			> 20	185(0.8)	0(0.0)	0 : 185	0(0.0)	0 : 185	0(0.0)	0 (0.0)	0:185
6	Damad	90,827	0–5	31,87(3.5)	3(42.9)	1:1,062	0(0.0)	0 : 3,187	2(6.9)	5 (45.5)	1 : 637
			6–10	6,117(6.7)	4(57.1)	1:1,529	1(100.0)	1:6,117	1(3.4)	6 (54.5)	1 : 1,020
			10–15	11,528 (12.7)	0(0.0)	0 : 11,528	0(0.0)	0 : 11,528	0(0.0)	0 (0.0)	0 : 11,528
			> 20	69,995 (77.1)	0(0.0)	0 : 69,995	0(0.0)	0 : 69,995	0(0.0)	0 (0.0)	0 : 69,995
7	Ar Rayth	23,543	0–5	55 (0.2)	0(0.0)	0 : 55	1(100.0)	1: 55	0(0.0)	1 (3.33)	1 : 55
			6–10	77 (0.3)	1(50.0)	1:77	0(0.0)	0 : 77	0(0.0)	1 (3.33)	1 : 77
			10–15	188(0.8)	1(50.0)	1:188	0(0.0)	0 : 188	0(0.0)	1 (3.33)	1:188
			> 20	23,223 (98.6)	0(0.0)	0 : 23,223	0(0.0)	0 : 23,223	0(0.0)	0 (0.0)	0 : 23,223
8	Baysh	89,219	0–5	3 (0.0)	0(0.0)	0 : 3	0(0.0)	0 : 3	1(11.1)	1 (5.3)	1:3
			6–10	225(0.3)	3(33.3)	1:75	0(0.0)	0 : 225	0(0.0)	3 (15.8)	1: 75
			10–15	3,221(3.6)	6(66.7)	1:536	1(100.0)	1: 3,221	0(0.0)	7 (36.8)	1 : 460
			> 20	85,770 (96.1)	0(0.0)	0 : 85,770	0(0.0)	0 : 85,770	8(88.9)	8 (42.1)	1 :10,721
9	Farasan	22,556	0–5	0 (0.0)	0(0.0)	0 :0	0(0.0)	0 : 0	0(0.0)	0 (0.0)	0 : 0
			6–10	402(1.8)	1(33.3)	1:402	0(0.0)	0 : 402	0(0.0)	1 (25.0)	1 : 402
			10–15	402 (1.8)	0(0.0)	0 : 402	0(0.0)	0 : 402	0(0.0)	0 (0.0)	0 : 402
			> 20	21,752 (96.4)	2(66.7)	1:10,876	1(100.0)	1:21,752	0(0.0)	3 (75.0)	1 : 7,250
10	Ad Dair	73,087	0–5	0 (0.0)	0(0.0)	0 :0	0(0.0)	0 : 0	0(0.0)	0 (0.0)	0 : 0
			6–10	264(0.4)	2(22.2)	1:132	0(0.0)	0 : 264	3(100.0)	5 (38.5)	1 :53
			10–15	399 (0.5)	4(44.4)	1: 99.75	1(100.0)	1: 399	0(0.0)	5 (38.5)	1: 80
			> 20	72,424(99.1)	3(33.3)	1:24,141	0(0.0)	0 : 72,424	0(0.0)	3 (23.1)	1 :24,141
11	Ahad Al Masarihah	139,498	0–5	12,191(8.7)	0(0.0)	0 : 12,191	0(0.0)	0 : 12,191	0(0.0)	0 (0.0)	0 :12,191
			6–10	25,048 (18.0)	2(16.7)	1:12,524	0(0.0)	0 : 25,048	0(0.0)	2 (10.5)	1 :12,524
			10–15	51,238(36.7)	6(50.0)	1:8,539	1(100.0)	1:51,238	6(100.0)	13 (68.4)	1 :3,941
			> 20	51,021 (36.6)	4(33.3)	1:12,755	0(0.0)	0 : 12,755	0(0.0)	4 (21.1)	1 : 12,755
12	Al Aydabi	40,106	0–5	82(0.2)	1(25.0)	1:82	0(0.0)	0 : 82	0(0.0)	1 (12.8)	1 : 82
			6–10	1,152 (2.9)	1(25.0)	1:1,152	0(0.0)	0 :1,152	2(66.7)	3 (37.5)	1 : 384
			10–15	3,000 (7.5)	2(50.0)	1:1,500	1(100.0)	1:3,000	1(33.3)	4 (50.0)	1 : 750
			> 20	35,872 (89.4)	0(0.0)	0 : 35,872	0(0.0)	0 : 35,872	0(0.0)	0 (0.0)	0 : 35,872
13	Al Aridah	96,420	0–5	2,044 (2.1)	2(25.0)	1:1,022	1(100.0)	1:2,044	3(100)	6 (50.0)	1 :340
			6–10	5,131(5.3)	2(25.0)	1:2,565	0(0.0)	0 : 5,131	0(0.0)	2 (16.7)	1 :2,566
			10–15	7,575 (7.9)	4(50.0)	1:1,893	0(0.0)	0 : 7,575	0(0.0)	4 (33.3)	1 : 1,893
			> 20	81,670 (84.7)	0(0.0)	0 : 81,670	0(0.0)	0 : 81,670	0(0.0)	0 (0.0)	0 : 81,670
14	Ad Darb	87,932	0–5	2,484(2.8)	1(33.3)	1:2,484	0(0.0)	0 : 2,484	0(0.0)	1 (10.0)	1 :2,484
			6–10	13,019 (14.8)	0(0.0)	0 : 13,019	1(100.0)	1:13,019	4(66.7)	5 (50.0)	1 :2,604
			10–15	30,064 (34.2)	1(33.3)	30,064	0(0.0)	0 : 30,064	1(16.7)	2 (20.0)	1 : 15,032
			> 20	42,365 (48.2)	1(33.3)	42,365	0(0.0)	0 : 42,365	1(16.7)	2 (20.0)	1 : 12,182
15	At Tuwal	68,760	0–5	37 (0.1)	0(0.0)	0 : 37	0(0.0)	0 : 37	0(0.0)	0 (0.0)	0 :37
			6–10	10,786 (15.7)	2(40.0)	5,393	0(0.0)	0 : 10,786	0(0.0)	2 (33.3)	1 : 5,393
			10–15	27,707 (40.3)	3(60.0)	1:9,235	1(100.0)	1:27,707	0(0.0)	4 (66.7)	1 :6,926
			> 20	30,230 (44.0)	0(0.0)	0 : 27,707	0(0.0)	0 : 30,230	0(0.0)	0 (0.0)	0 :30,230
16	Harub	36,068	0–5	136 (0.4)	1(16.7)	1:136	0(0.0)	0 : 136	1(50.0)	2 (12.5)	1 :68
			6–10	833(2.3)	3(50.0)	1:277	0(0.0)	0 : 833	1(50.0)	4 (50.0)	1 : 208
			10–15	1,128 (3.1)	2(33.3)	1:564	0(0.0)	0 : 1,128	0(0.0)	2 (12.5)	1 :564
			> 20	33,971 (94.2)	0(0.0)	0 : 33,971	0(0.0)	0 : 33,971	0(0.0)	0 (0.0)	0 : 33,971
17	Fayfa	38,005	0–5	65 (0.2)	3(100.0)	1:21	1(100.0)	1:65	0(0.0)	4 (100.0)	1 :16
			6–10	1,506 (4.0)	0(0.0)	0 : 1,506	0(0.0)	0 : 1,506	0(0.0)	0 (0.0)	0 :1,506
			10–15	839 (2.2)	0(0.0)	0 : 839	0(0.0)	0 : 839	0(0.0)	0 (0.0)	0 :839
			> 20	35,595 (93.7)	0(0.0)	0 : 35,595	0(0.0)	0 : 35,595	0(0.0)	0 (0.0)	0 : 35,595
Total		1,726,739	0–5	56,880 (3.3)	28(19.3)	1:2,031	6(33.3)	1:9,480	58(51.8)	92 (33.5)	1 :618
			6–10	161,666 (9.4)	39(26.9)	1: 4,145	4(22.2)	1:40,416	18(16.1)	61 (22.2)	1 : 2,650
			10–15	305,772 (17.7)	57(39.3)	1: 5,364	7(38.9)	1:43,681	24(21.4)	88 (32.0)	1 : 3,475
			> 20	120,2421 (69.6)	21(14.5)	1: 57,258	1(5.6)	1:120,2421	12(10.7)	34 (12.4)	1 : 35,365

Abbreviation: PHCs: Primary Healthcare Centres, n: number

The results showed that Jazan, Sabya, Abu Arish, and Samtah had the highest number of dental clinics and were also the most populous governorates. However, only 6.6%,3.6% and 7.0% of Jazan, Abu Arish, and Samtah governorates had access to 72.0%, 48.5% and 51.7%, respectively, of the dental clinics within 5 km of the governorate’s centres. More than a third (65.9%) of the dental clinics were located within 15 km of the Sabya governorate centre, which provided dental services to only 4.9% of the population (Table 2).

**Fig. 2:**
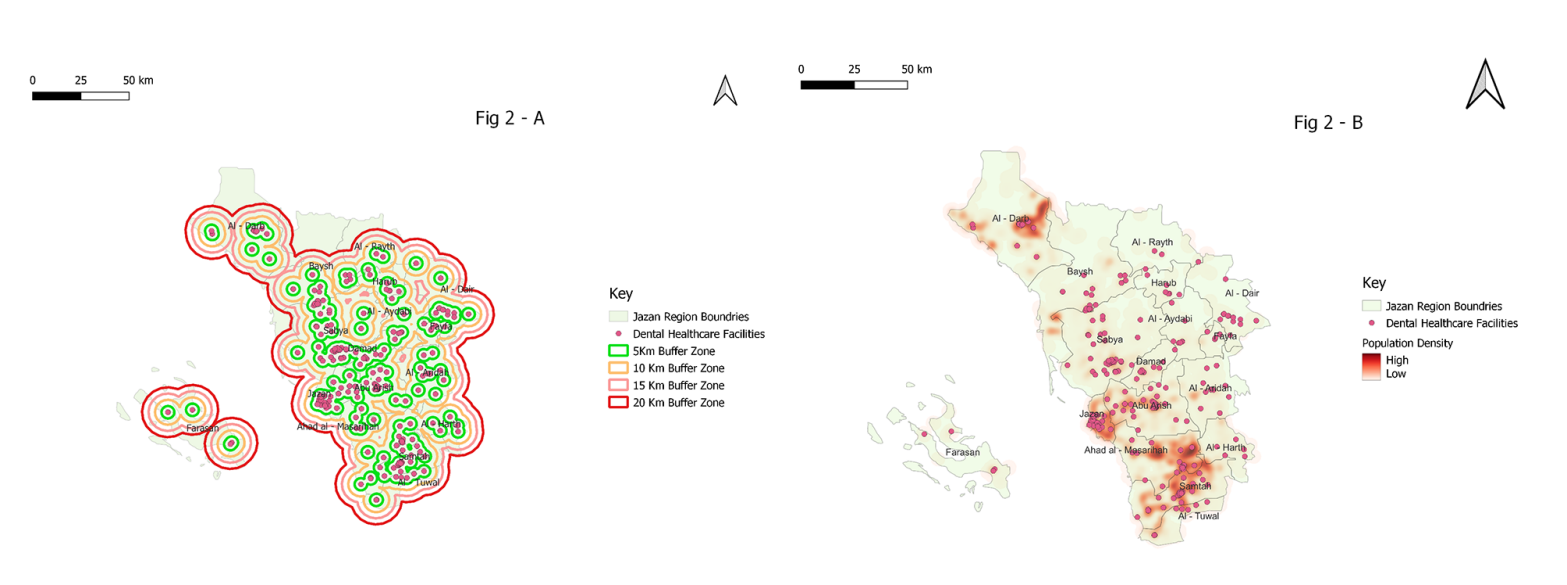
A high-resolution image showing the distribution of population per governorate and locations of dental clinics, (a) with dental clinics being used as the centroid of the buffer; (b) heat map indicating the distribution of population density and dental clinic in Jazan region

When analysed from the perspective of dental clinic centroids, Governorates Damad, Ad Dair, Ahad Al Masarihah, and Al Aridah had the lowest accessibility to dental healthcare, indicating that they were served by dental clinics in neighbouring governorates. Significant clustering of dental clinic locations was observed in Jazan, Sabya, Samtah, and Abu Arish. Additionally, there are significant underserved areas in the peripheral regions of Baysh, Ar Rayth, Ad Darb, and Al Harth governorates (Fig. 2).

## Discussion

This study utilized GIS-based methods to investigate the distribution of public and private dental clinics in the Jazan region of KSA. The findings of this study may be useful for researchers and policymakers who are interested in understanding dental service availability and geographical accessibility issues in the region. The results of the study support the hypothesis that the distribution of dental clinics is positively correlated with population density. However, the study also found that the distribution of health facilities in the region is uneven as noted by Shubayr et al. [11], which may impact access to dental care for residents.

Over one-third of the region’s population resides in major governorates such as Sabya and Abu Arish, where a greater number of dental clinics are present but in less accessible areas. The dental clinic-to-population ratios in Sabya and Abu Arish are 1:7,079 and 1:7,535, respectively, which are lower than the ratios found in Alsharif’s study [9]. Alsharif’s study found that highly populated areas with a greater number of dental clinics had high practice-to-population ratios, with ratios of 1:15,514 and 1:27,949 in highly accessible and accessible areas, respectively.

According to a study conducted in Jazan, the ratio of MOH- affiliated dentists to people in the Jazan region is 1:6,600 [23]. However, when considering public and private dental clinics, as reported in the MOH statistical yearly book [17], the ratio in Jazan is 1:2422, similar to the ratio in the Eastern region (1:2477) and higher than the ratios in Riyadh and Jeddah, which are 1:1522 and 1:1688 respectively and also higher than the ratios found in the USA (1:1,639) [24], Netherlands (1:1818) [25], and the UK (1:1886) [26]. The study also found that there is only one dental clinic for every 6,279 people in the Jazan region, indicating a heavy burden on the region’s government-run public dental facilities. his shortage of dental clinics suggests that many people may not receive preventive, basic, or advanced dental treatments, which could affect their oral health status, and the high demand on public health facilities makes accessing these services difficult [27].

Improving the distribution of dental clinics is crucial for ensuring convenient access to dental care. While the majority of dental clinics are located within 5 and 15 km of the governorate’s centre, a few are situated beyond a 20 km radius from the centre, posing challenges in accessing dental care, particularly in rural areas. This finding is consistent with studies conducted in Jeddah [14] and Jazan [23], which found that several peripheral areas were underserved by healthcare facilities. Consequently, central areas are likely to be more crowded than other areas, leading to longer waiting times and far appointments. Moreover, only 3.3% of the population lives within 5 km of a governorate centre, while 70% of the population is located beyond the 20 km buffer, making it difficult for them to access dental clinics and resulting in the population in these areas having to travel long distances to access dental clinics [28]. This difficulty in accessing dental care is a significant barrier to obtaining dental services, particularly for those living in peripheral areas where spatial access to dental care is generally lower than in central or inner areas. This aligns with findings from various countries, including KSA, Australia, Brazil, and New Zealand [9, 27, 29–31], which have also identified that people living in peripheral areas have less spatial access to dental care compared to those living in central or inner areas.

The study found that governorates in the Jazan region, including Al Harth, Fayfa, and Atwal, have a low percentage of dental facilities, which is consistent with a study that found few dental providers in the area [11]. As a result, residents may need to travel to the nearest area and overload some MOH facilities, resulting in lower quality of service. A similar study discovered that many residents of the Jazan governorate may need to drive for about 30 min to reach MOH PHCs. Moreover, the mountainous governorates of Al Aridah, Al Aydabi, Al Harth, Ar Rayth, Baysh, Fayfa, and Harub were found to have a large population with limited access to MOH PHCs [23]. This lack of access is likely due to the challenging driving conditions in these mountainous regions [23]. Dental clinics also underserve these mountainous and peripheral regions of the governorates.

A contemporary dental healthcare system is needed to address the inadequacies of the current system [9]. This requires direct interaction between dentists and patients, improved data collection, storage, and exchange, particularly for populations in remote locations [32]. Public-private partnerships have been proposed as a way to make the healthcare system more flexible and efficient, addressing common goals and meeting the needs of the population [33]. In particular, the dental healthcare system in the Jazan region needs improvement, especially in peripheral governorates such as Al Harth, Fayfa, Altuwal, and Farasan, which currently have few public dental clinic and no private dental clinics. To improve access to dental healthcare services in these areas, several solutions could be considered, including provide additional training and consultation to non-dental health providers. This can enable them to provide basic preventive and diagnostic services and refer patients who require specialized dental care to the closest dental clinics. In addition, implementing compulsory internship programs for graduated dental students in rural or underserved areas in the region, and supporting rural area residents to be treated in the closest private dental clinics [34].

This study is the first to use GIS to examine the distribution of public and private dental healthcare services in Jazan region. However, several limitations should be considered when interpreting the results. The study did not have data on the type of oral health services, facilities, and providers, particularly in the private sector in the Jazan region, as well as on their accessibility, characteristics (such as gender and specialization), and the number of oral health providers (dentists, dental hygienists, and assistants) per district population. Additionally, the oral health system and healthcare system are constantly evolving, so the data obtained in this study may not reflect more recent changes.

To address these issues, it is necessary to analyse the types of dental facilities, service availability (including opening hours), workforce numbers per facility, and the treatments provided by dental clinics in the region. This will help to identify the specific needs and challenges of the dental healthcare system in Jazan. To further validate these findings, it would be helpful to map public, private, and other health facilities in the region, as well as assess the burden of oral health issues in the area. Overall, these steps could help to improve the distribution and accessibility of dental healthcare services in Jazan region, ensuring that residents have access to the care they need.

## Conclusion

The current dental healthcare system in Jazan region is inadequate, and a shift towards a contemporary system is needed to improve access and distribution of dental healthcare services. Public-private partnerships have been proposed as a potential solution, and increased focus on serving peripheral and mountainous areas of the region is necessary. The findings of this study emphasize the need for improvements in the distribution of dental healthcare services in the Jazan region, and efforts are needed to map health facilities and assess the burden of oral diseases in the region to validate the findings. Improving the distribution and accessibility of dental healthcare services in Jazan region is crucial to ensure that residents have access to the care they need, and to improve their oral health status.

## Data Availability

The population datasets analysed in this study are available on the General Authority [https://www.stats.gov.sa/en/1007-0] and World Population’s [https://www.worldpop.org/] websites. The location of health facilities was obtained from the MOH Statistical Yearbook [https://www.moh.gov.sa/en/Ministry/Statistics/book/Pages/default.aspx] and a Ministry of Health interactive map [https://www.moh.gov.sa/en/eServices/interactive-maps/Pages/default.aspx#/].
